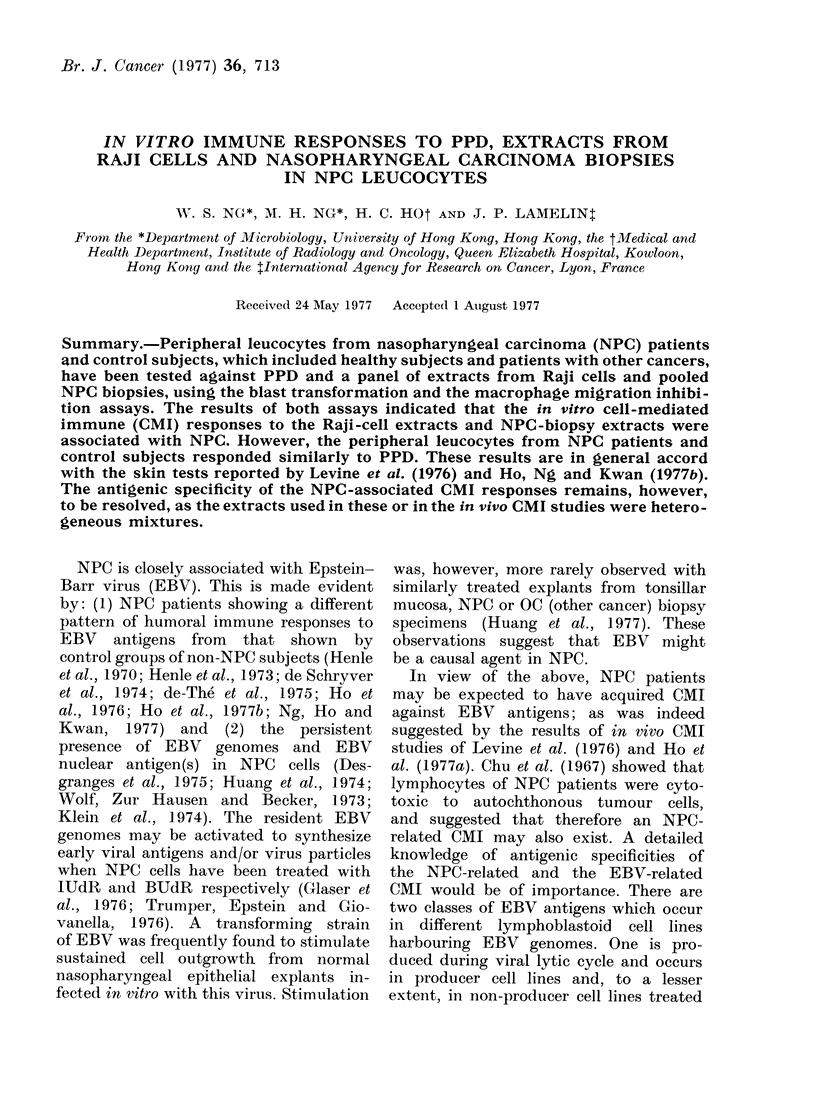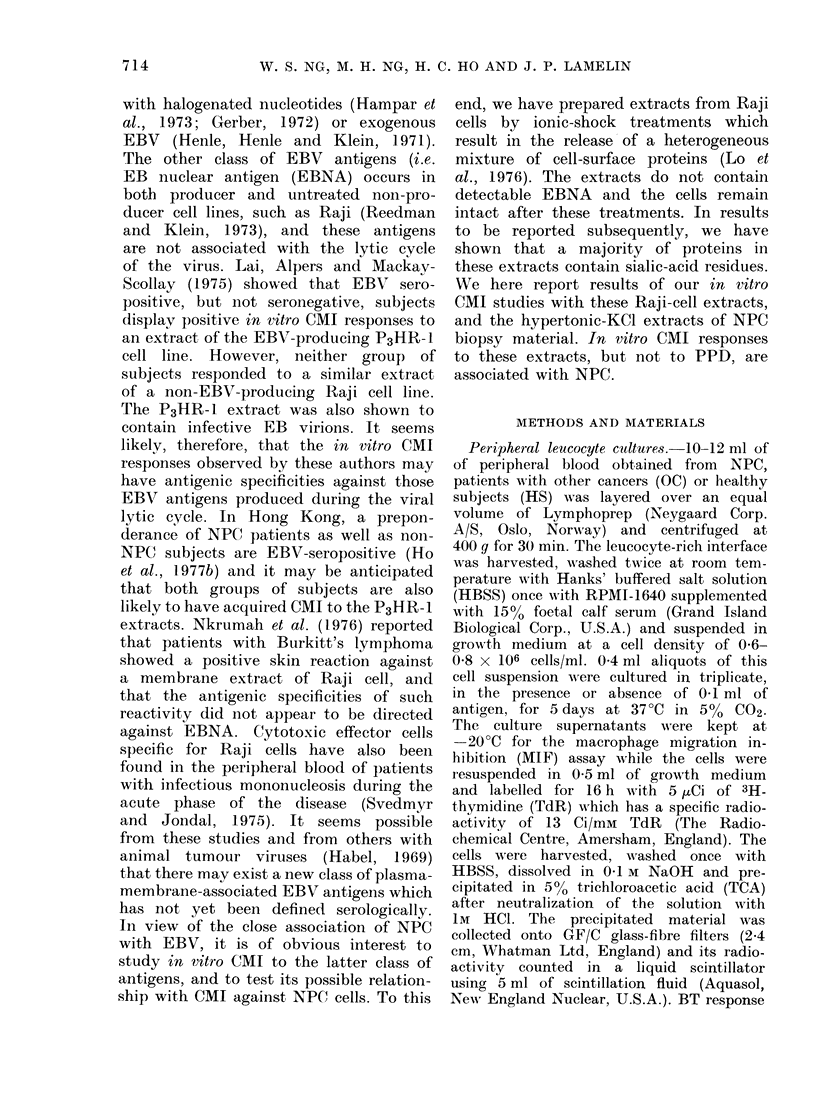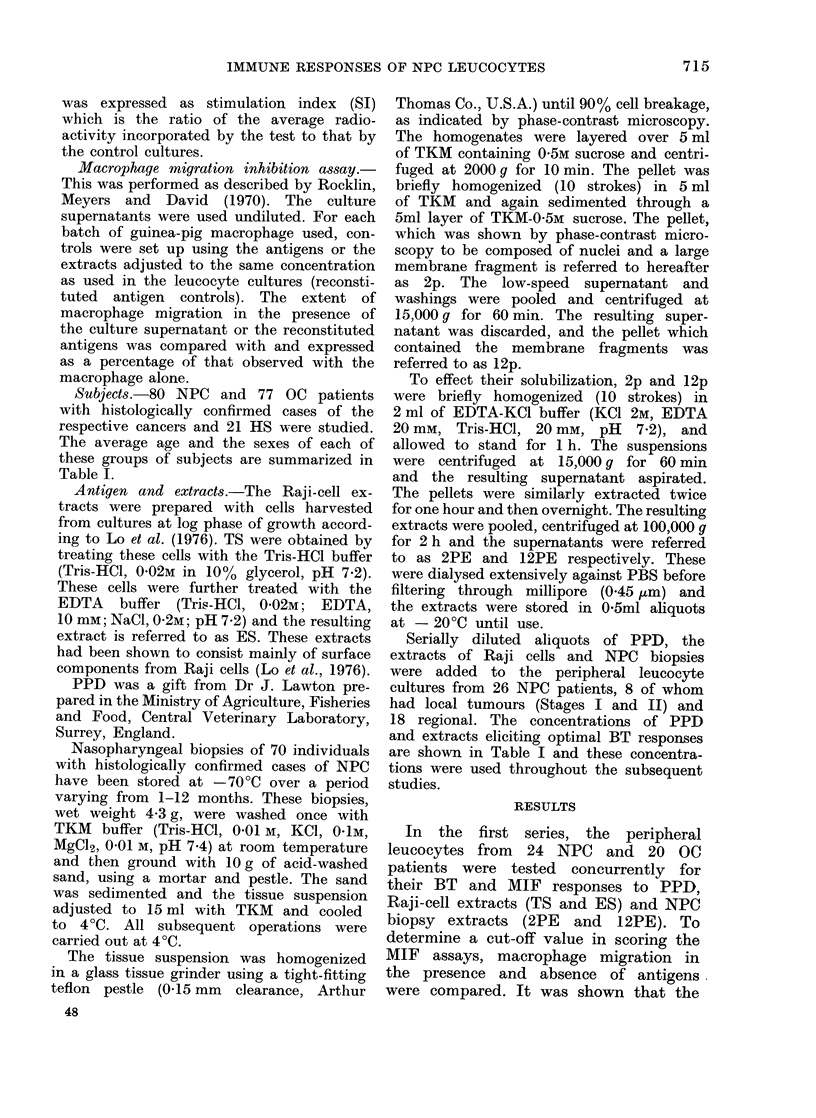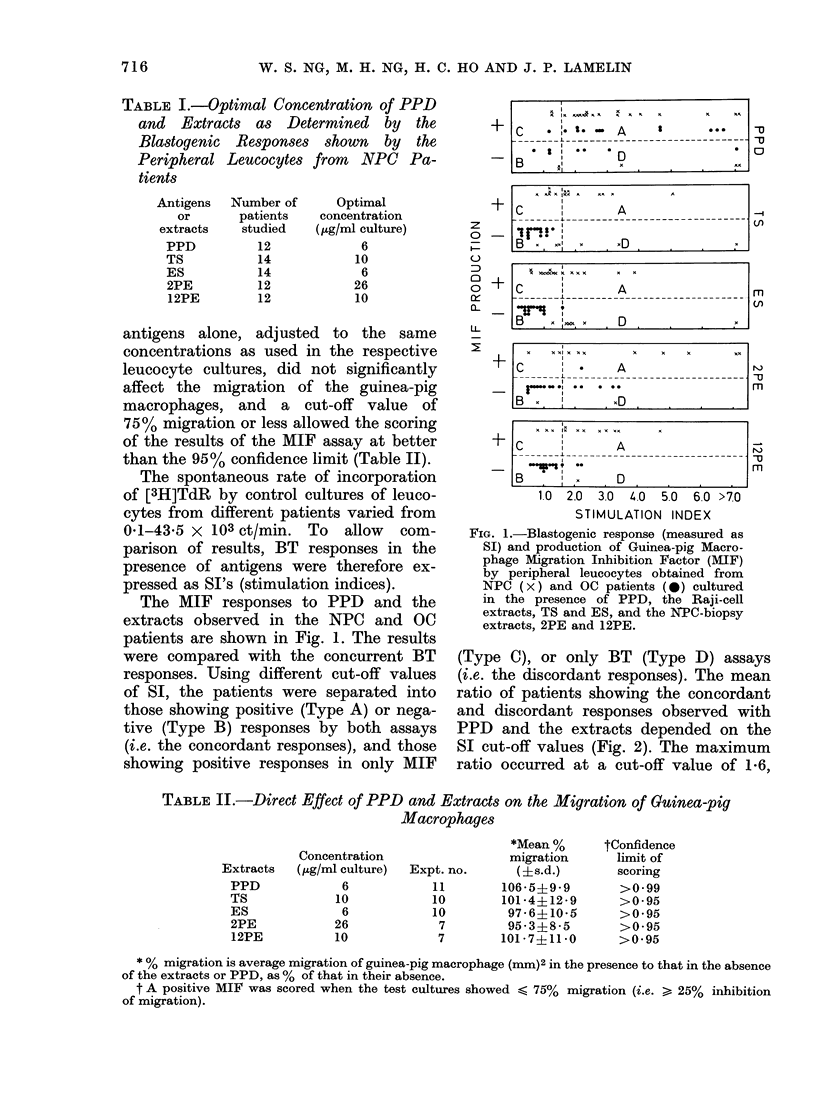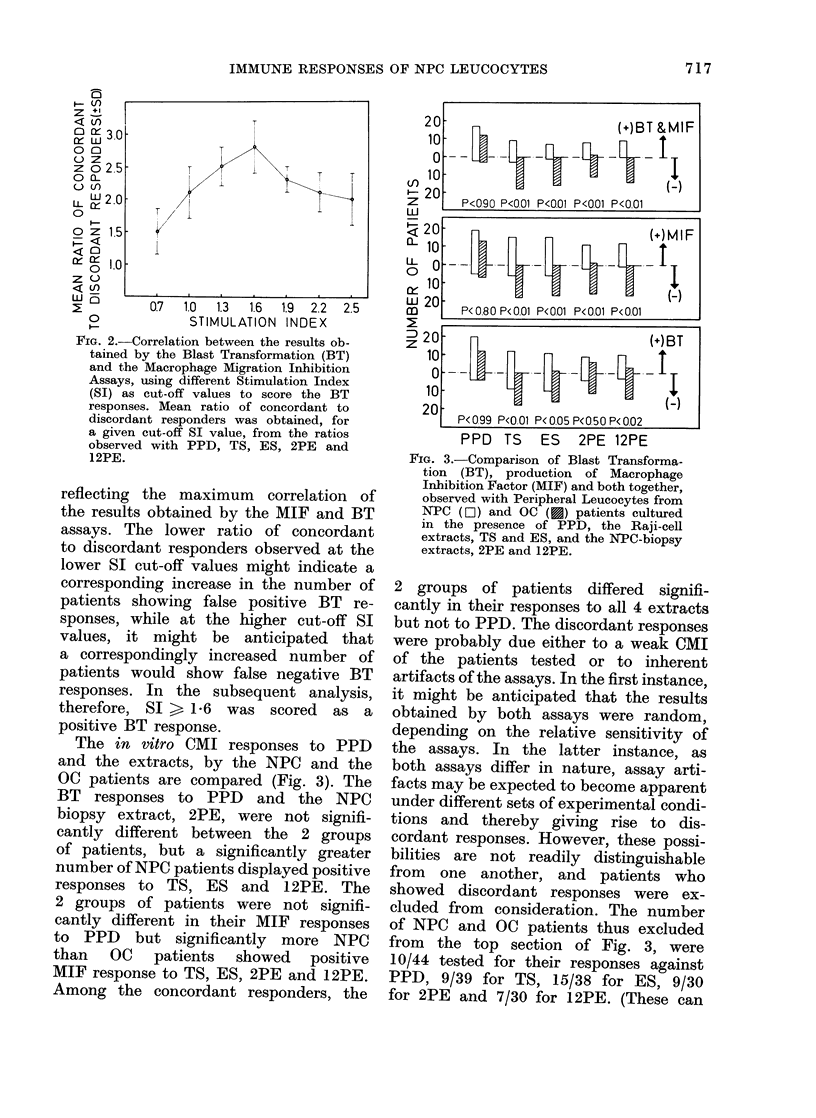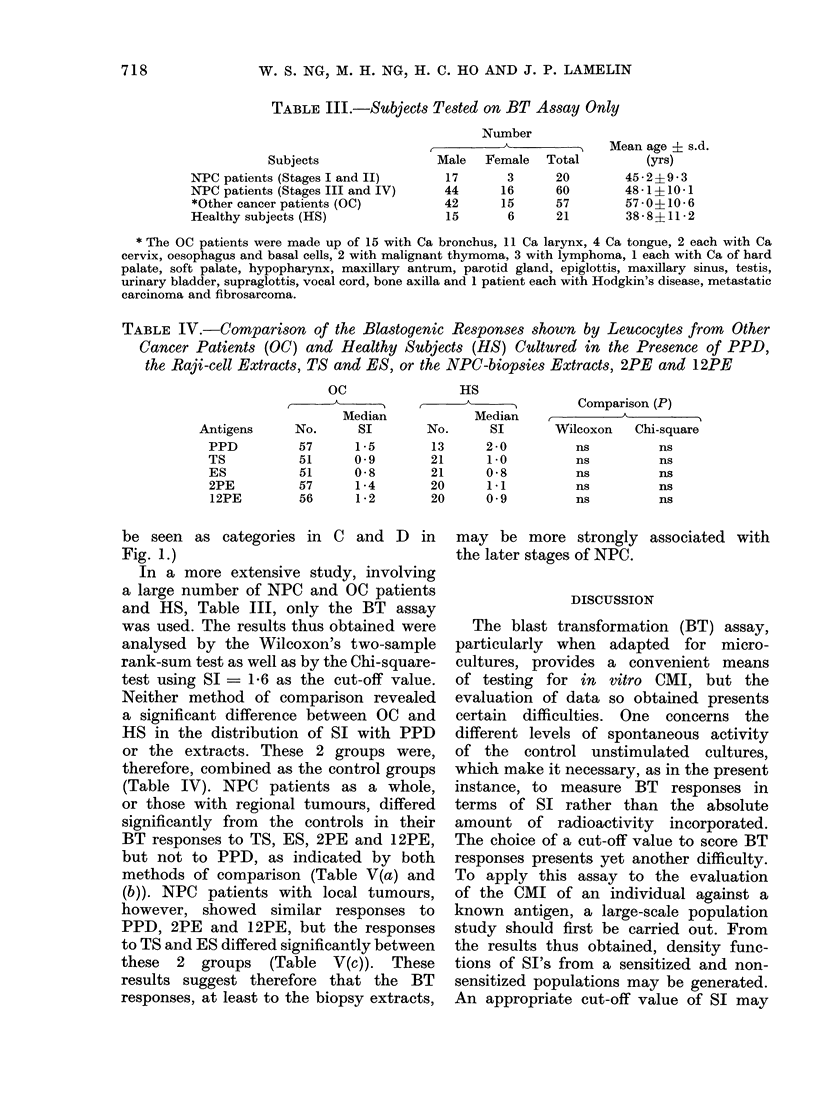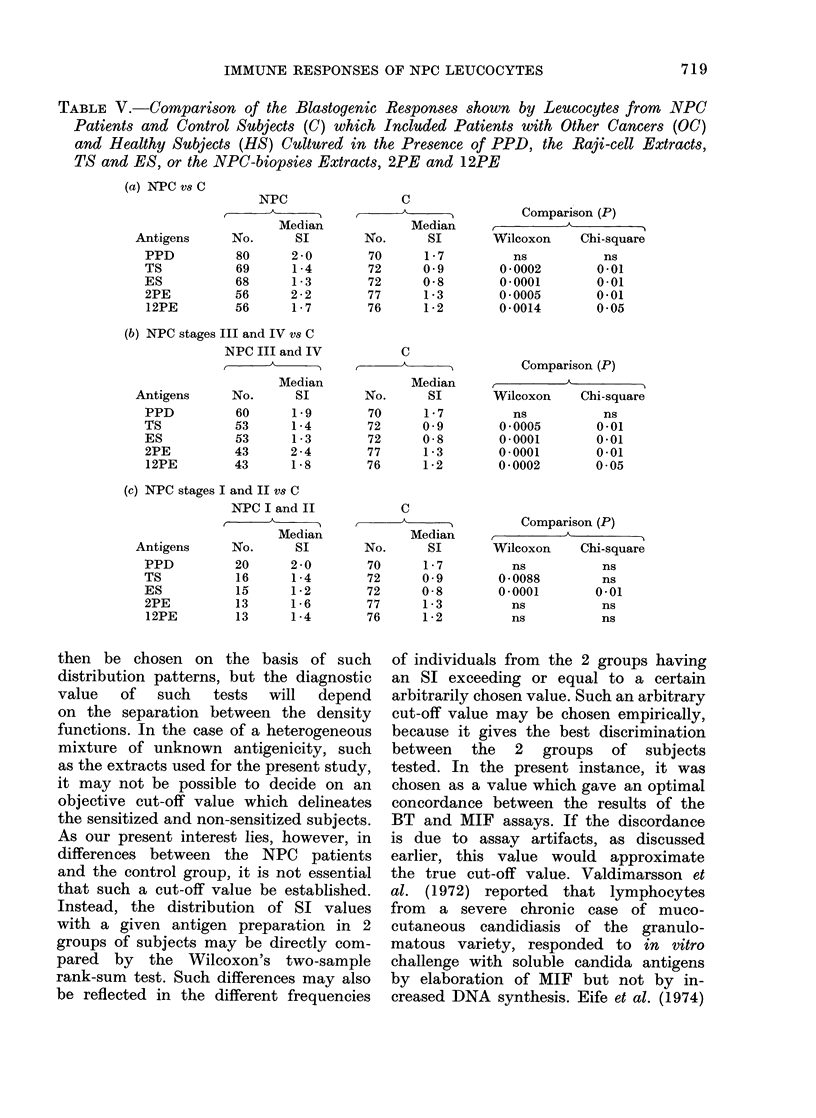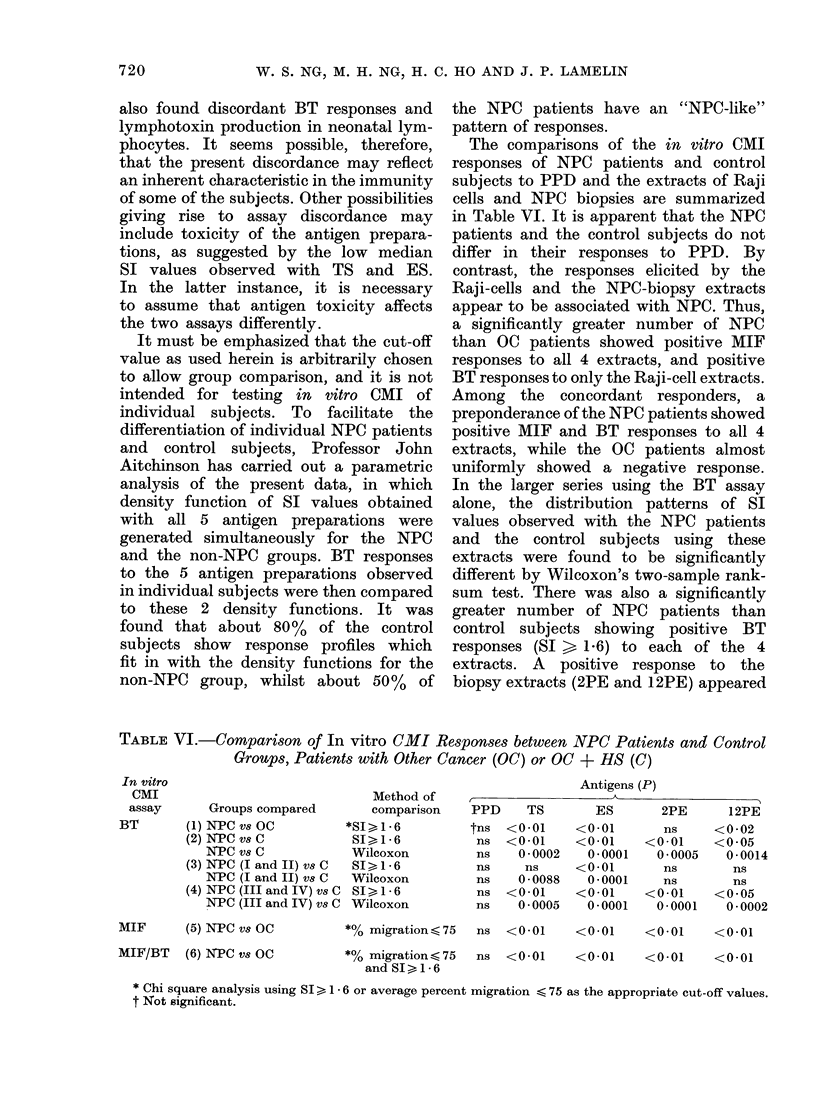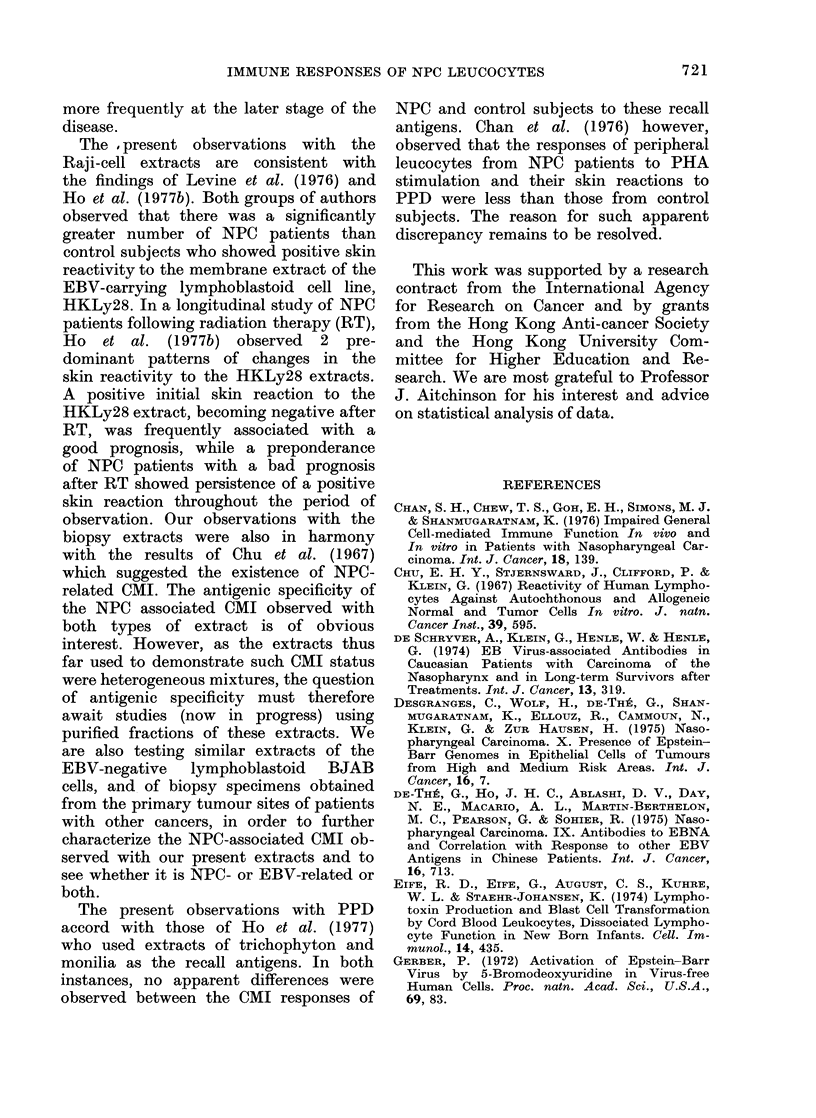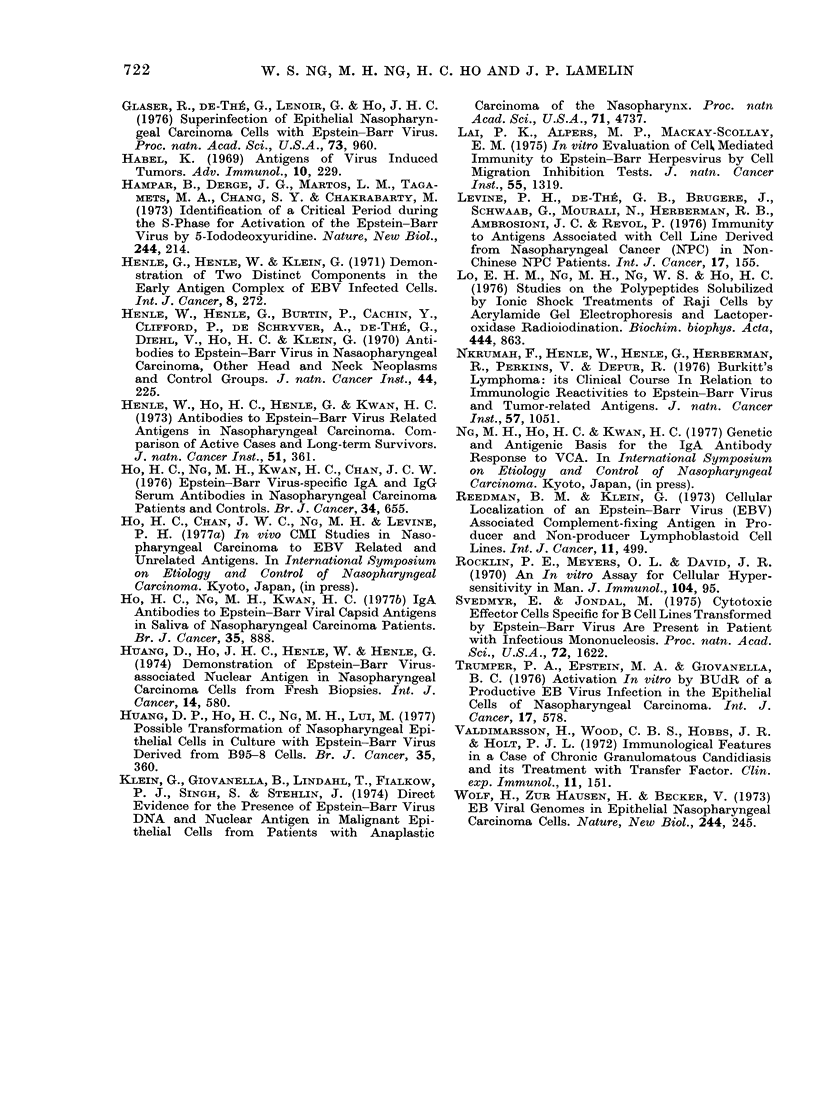# In vitro immune responses to PPD, extracts from Raji cells and nasopharyngeal carcinoma biopsies in NPC leucocytes.

**DOI:** 10.1038/bjc.1977.254

**Published:** 1977-12

**Authors:** W. S. Ng, M. H. Ng, H. C. Ho, J. P. Lamelin

## Abstract

Peripheral leucocytes from nasopharyngeal carcinoma (NPC) patients and control subjects, which included healthy subjects and patients with other cancers, have been tested against PPD and a panel of extracts from Raji cells and pooled NPC biopsies, using the blast transformation and the macrophage migration inhibition assays. The results of both assays indicated that the in vitro cell-mediated immune (CMI) responses to the Raji-cell extracts and NPC-biopsy extracts were associated with NPC. However, the peripheral leucocytes from NPC patients and control subjects responded similarly to PPD. These results are in general accord with the skin tests reported by Levine et al. (1976) and Ho, Ng and Kwan (1977b). The antigenic specificity of the NPC-associated CMI responses remains, however, to be resolved, as the extracts used in these or in the in vivo CMI studies were heterogeneous mixtures.


*Br. J. Cancer* (1977) **36**, 713

### *IN VITRO* IMMUNE RESPONSES TO PPD, EXTRACTS FROM RAJI CELLS AND NASOPHARYNGEAL CARCINOMA BIOPSIES IN NPC LEUCOCYTES

W. S. NG*, M. H. NG*, H. C. HO† AND J. P. LAMELIN‡

*From the *Department of Microbiology, University of Hong Kong, Hong Kong, the †Medical and Health Department, Institute of Radiology and Oncology, Queen Elizabeth Hospital, Kowloon, Hong Kong and the ‡International Agency for Research on Cancer, Lyon, France*

Received 24 May 1977 Accepted 1 August 1977

**Summary.—Peripheral leucocytes from nasopharyngeal carcinoma (NPC) patients and control subjects, which included healthy subjects and patients with other cancers, have been tested against PPD and a panel of extracts from Raji cells and pooled NPC biopsies, using the blast transformation and the macrophage migration inhibition assays. The results of both assays indicated that the *in vitro* cell-mediated immune (CMI) responses to the Raji-cell extracts and NPC-biopsy extracts were associated with NPC. However, the peripheral leucocytes from NPC patients and control subjects responded similarly to PPD. These results are in general accord with the skin tests reported by Levine *et al.* (1976) and Ho, Ng and Kwan (1977*b*). The antigenic specificity of the NPC-associated CMI responses remains, however, to be resolved, as the extracts used in these or in the *in vivo* CMI studies were heterogeneous mixtures.**

NPC is closely associated with Epstein–Barr virus (EBV). This is made evident by: (1) NPC patients showing a different pattern of humoral immune responses to EBV antigens from that shown by control groups of non-NPC subjects (Henle *et al.*, 1970; Henle *et al.*, 1973; de Schryver *et al.*, 1974; de-Thé *et al.*, 1975; Ho *et al.*, 1976; Ho *et al.*, 1977*b*; Ng, Ho and Kwan, 1977) and (2) the persistent presence of EBV genomes and EBV nuclear antigen(s) in NPC cells (Desgranges *et al.*, 1975; Huang *et al.*, 1974; Wolf, Zur Hausen and Becker, 1973; Klein *et al.*, 1974). The resident EBV genomes may be activated to synthesize early viral antigens and/or virus particles when NPC cells have been treated with IUdR and BUdR respectively (Glaser *et al.*, 1976; Trumper, Epstein and Giovanella, 1976). A transforming strain of EBV was frequently found to stimulate sustained cell outgrowth from normal nasopharyngeal epithelial explants infected *in vitro* with this virus. Stimulation was, however, more rarely observed with similarly treated explants from tonsillar mucosa, NPC or OC (other cancer) biopsy specimens (Huang *et al.*, 1977). These observations suggest that EBV might be a causal agent in NPC.

In view of the above, NPC patients may be expected to have acquired CMI against EBV antigens; as was indeed suggested by the results of *in vivo* CMI studies of Levine *et al.* (1976) and Ho *et al.* (1977*a*). Chu *et al.* (1967) showed that lymphocytes of NPC patients were cytotoxic to autochthonous tumour cells, and suggested that therefore an NPC-related CMI may also exist. A detailed knowledge of antigenic specificities of the NPC-related and the EBV-related CMI would be of importance. There are two classes of EBV antigens which occur in different lymphoblastoid cell lines harbouring EBV genomes. One is produced during viral lytic cycle and occurs in producer cell lines and, to a lesser extent, in non-producer cell lines treated with halogenated nucleotides (Hampar *et al.*, 1973; Gerber, 1972) or exogenous EBV (Henle, Henle and Klein, 1971). The other class of EBV antigens (*i.e.* EB nuclear antigen (EBNA) occurs in both producer and untreated non-producer cell lines, such as Raji (Reedman and Klein, 1973), and these antigens are not associated with the lytic cycle of the virus. Lai, Alpers and Mackay-Scollay (1975) showed that EBV seropositive, but not seronegative, subjects display positive *in vitro* CMI responses to an extract of the EBV-producing P3HR-1 cell line. However, neither group of subjects responded to a similar extract of a non-EBV-producing Raji cell line. The P3HR-1 extract was also shown to contain infective EB virions. It seems likely, therefore, that the *in vitro* CMI responses observed by these authors may have antigenic specificities against those EBV antigens produced during the viral lytic cycle. In Hong Kong, a preponderance of NPC patients as well as non-NPC subjects are EBV-seropositive (Ho *et al.*, 1977*b*) and it may be anticipated that both groups of subjects are also likely to have acquired CMI to the P3HR-1 extracts. Nkrumah *et al.* (1976) reported that patients with Burkitt's lymphoma showed a positive skin reaction against a membrane extract of Raji cell, and that the antigenic specificities of such reactivity did not appear to be directed against EBNA. Cytotoxic effector cells specific for Raji cells have also been found in the peripheral blood of patients with infectious mononucleosis during the acute phase of the disease (Svedmyr and Jondal, 1975). It seems possible from these studies and from others with animal tumour viruses (Habel, 1969) that there may exist a new class of plasma-membrane-associated EBV antigens which has not yet been defined serologically. In view of the close association of NPC with EBV, it is of obvious interest to study *in vitro* CMI to the latter class of antigens, and to test its possible relationship with CMI against NPC cells. To this end, we have prepared extracts from Raji cells by ionic-shock treatments which result in the release of a heterogeneous mixture of cell-surface proteins (Lo *et al.*, 1976). The extracts do not contain detectable EBNA and the cells remain intact after these treatments. In results to be reported subsequently, we have shown that a majority of proteins in these extracts contain sialic-acid residues. We here report results of our *in vitro* CMI studies with these Raji-cell extracts, and the hypertonic-KCl extracts of NPC biopsy material. *In vitro* CMI responses to these extracts, but not to PPD, are associated with NPC.

### METHODS AND MATERIALS

*Peripheral leucocyte cultures.*—10–12 ml of of peripheral blood obtained from NPC, patients with other cancers (OC) or healthy subjects (HS) was layered over an equal volume of Lymphoprep (Neygaard Corp. A/S, Oslo, Norway) and centrifuged at 400 *g* for 30 min. The leucocyte-rich interface was harvested, washed twice at room temperature with Hanks' buffered salt solution (HBSS) once with RPMI-1640 supplemented with 15% foetal calf serum (Grand Island Biological Corp., U.S.A.) and suspended in growth medium at a cell density of 0·6–0·8 × $10^6$ cells/ml. 0·4 ml aliquots of this cell suspension were cultured in triplicate, in the presence or absence of 0·1 ml of antigen, for 5 days at 37°C in 5% $CO_2$. The culture supernatants were kept at −20°C for the macrophage migration inhibition (MIF) assay while the cells were resuspended in 0·5 ml of growth medium and labelled for 16 h with 5 $\mu$Ci of $^3$H-thymidine (TdR) which has a specific radioactivity of 13 Ci/mM TdR (The Radiochemical Centre, Amersham, England). The cells were harvested, washed once with HBSS, dissolved in 0·1 M NaOH and precipitated in 5% trichloroacetic acid (TCA) after neutralization of the solution with 1M HCl. The precipitated material was collected onto GF/C glass-fibre filters (2·4 cm, Whatman Ltd, England) and its radioactivity counted in a liquid scintillator using 5 ml of scintillation fluid (Aquasol, New England Nuclear, U.S.A.). BT response was expressed as stimulation index (SI) which is the ratio of the average radioactivity incorporated by the test to that by the control cultures.

*Macrophage migration inhibition assay.*—This was performed as described by Rocklin, Meyers and David (1970). The culture supernatants were used undiluted. For each batch of guinea-pig macrophage used, controls were set up using the antigens or the extracts adjusted to the same concentration as used in the leucocyte cultures (reconstituted antigen controls). The extent of macrophage migration in the presence of the culture supernatant or the reconstituted antigens was compared with and expressed as a percentage of that observed with the macrophage alone.

*Subjects.*—80 NPC and 77 OC patients with histologically confirmed cases of the respective cancers and 21 HS were studied. The average age and the sexes of each of these groups of subjects are summarized in Table I.

*Antigen and extracts.*—The Raji-cell extracts were prepared with cells harvested from cultures at log phase of growth according to Lo *et al.* (1976). TS were obtained by treating these cells with the Tris-HCl buffer (Tris-HCl, 0·02M in 10% glycerol, pH 7·2). These cells were further treated with the EDTA buffer (Tris-HCl, 0·02M; EDTA, 10 mM; NaCl, 0·2M; pH 7·2) and the resulting extract is referred to as ES. These extracts had been shown to consist mainly of surface components from Raji cells (Lo *et al.*, 1976).

PPD was a gift from Dr J. Lawton prepared in the Ministry of Agriculture, Fisheries and Food, Central Veterinary Laboratory, Surrey, England.

Nasopharyngeal biopsies of 70 individuals with histologically confirmed cases of NPC have been stored at −70°C over a period varying from 1–12 months. These biopsies, wet weight 4·3 g, were washed once with TKM buffer (Tris-HCl, 0·01 M, KCl, 0·1M, $MgCl_2$, 0·01 M, pH 7·4) at room temperature and then ground with 10 g of acid-washed sand, using a mortar and pestle. The sand was sedimented and the tissue suspension adjusted to 15 ml with TKM and cooled to 4°C. All subsequent operations were carried out at 4°C.

The tissue suspension was homogenized in a glass tissue grinder using a tight-fitting teflon pestle (0·15 mm clearance, Arthur Thomas Co., U.S.A.) until 90% cell breakage, as indicated by phase-contrast microscopy. The homogenates were layered over 5 ml of TKM containing 0·5M sucrose and centrifuged at 2000 *g* for 10 min. The pellet was briefly homogenized (10 strokes) in 5 ml of TKM and again sedimented through a 5ml layer of TKM-0·5M sucrose. The pellet, which was shown by phase-contrast microscopy to be composed of nuclei and a large membrane fragment is referred to hereafter as 2p. The low-speed supernatant and washings were pooled and centrifuged at 15,000 *g* for 60 min. The resulting supernatant was discarded, and the pellet which contained the membrane fragments was referred to as 12p.

To effect their solubilization, 2p and 12p were briefly homogenized (10 strokes) in 2 ml of EDTA-KCl buffer (KCl 2M, EDTA 20 mM, Tris-HCl, 20 mM, pH 7·2), and allowed to stand for 1 h. The suspensions were centrifuged at 15,000 *g* for 60 min and the resulting supernatant aspirated. The pellets were similarly extracted twice for one hour and then overnight. The resulting extracts were pooled, centrifuged at 100,000 *g* for 2 h and the supernatants were referred to as 2PE and 12PE respectively. These were dialysed extensively against PBS before filtering through millipore (0·45 μm) and the extracts were stored in 0·5ml aliquots at − 20°C until use.

Serially diluted aliquots of PPD, the extracts of Raji cells and NPC biopsies were added to the peripheral leucocyte cultures from 26 NPC patients, 8 of whom had local tumours (Stages I and II) and 18 regional. The concentrations of PPD and extracts eliciting optimal BT responses are shown in Table I and these concentrations were used throughout the subsequent studies.

### RESULTS

In the first series, the peripheral leucocytes from 24 NPC and 20 OC patients were tested concurrently for their BT and MIF responses to PPD, Raji-cell extracts (TS and ES) and NPC biopsy extracts (2PE and 12PE). To determine a cut-off value in scoring the MIF assays, macrophage migration in the presence and absence of antigens were compared. It was shown that the

TABLE I.—*Optimal Concentration of PPD and Extracts as Determined by the Blastogenic Responses shown by the Peripheral Leucocytes from NPC Patients*

| Antigens or extracts | Number of patients studied | Optimal concentration (μg/ml culture) |
|---|---|---|
| PPD | 12 | 6 |
| TS | 14 | 10 |
| ES | 14 | 6 |
| 2PE | 12 | 26 |
| 12PE | 12 | 10 |

antigens alone, adjusted to the same concentrations as used in the respective leucocyte cultures, did not significantly affect the migration of the guinea-pig macrophages, and a cut-off value of 75% migration or less allowed the scoring of the results of the MIF assay at better than the 95% confidence limit (Table II).

The spontaneous rate of incorporation of [$^3$H]TdR by control cultures of leucocytes from different patients varied from 0·1–43·5 × $10^3$ ct/min. To allow comparison of results, BT responses in the presence of antigens were therefore expressed as SI's (stimulation indices).

The MIF responses to PPD and the extracts observed in the NPC and OC patients are shown in Fig. 1. The results were compared with the concurrent BT responses. Using different cut-off values of SI, the patients were separated into those showing positive (Type A) or negative (Type B) responses by both assays (*i.e.* the concordant responses), and those showing positive responses in only MIF (Type C), or only BT (Type D) assays (*i.e.* the discordant responses). The mean ratio of patients showing the concordant and discordant responses observed with PPD and the extracts depended on the SI cut-off values (Fig. 2). The maximum ratio occurred at a cut-off value of 1·6,

FIG. 1.—Blastogenic response (measured as SI) and production of Guinea-pig Macrophage Migration Inhibition Factor (MIF) by peripheral leucocytes obtained from NPC (×) and OC patients (●) cultured in the presence of PPD, the Raji-cell extracts, TS and ES, and the NPC-biopsy extracts, 2PE and 12PE.

TABLE II.—*Direct Effect of PPD and Extracts on the Migration of Guinea-pig Macrophages*

| Extracts | Concentration (μg/ml culture) | Expt. no. | *Mean % migration (±s.d.) | †Confidence limit of scoring |
|---|---|---|---|---|
| PPD | 6 | 11 | 106·5±9·9 | >0·99 |
| TS | 10 | 10 | 101·4±12·9 | >0·95 |
| ES | 6 | 10 | 97·6±10·5 | >0·95 |
| 2PE | 26 | 7 | 95·3±8·5 | >0·95 |
| 12PE | 10 | 7 | 101·7±11·0 | >0·95 |

* % migration is average migration of guinea-pig macrophage (mm)$^2$ in the presence to that in the absence of the extracts or PPD, as % of that in their absence.

† A positive MIF was scored when the test cultures showed ⩽ 75% migration (*i.e.* ⩾ 25% inhibition of migration).

FIG. 2.—Correlation between the results obtained by the Blast Transformation (BT) and the Macrophage Migration Inhibition Assays, using different Stimulation Index (SI) as cut-off values to score the BT responses. Mean ratio of concordant to discordant responders was obtained, for a given cut-off SI value, from the ratios observed with PPD, TS, ES, 2PE and 12PE.

FIG. 3.—Comparison of Blast Transformation (BT), production of Macrophage Inhibition Factor (MIF) and both together, observed with Peripheral Leucocytes from NPC (□) and OC (▨) patients cultured in the presence of PPD, the Raji-cell extracts, TS and ES, and the NPC-biopsy extracts, 2PE and 12PE.

reflecting the maximum correlation of the results obtained by the MIF and BT assays. The lower ratio of concordant to discordant responders observed at the lower SI cut-off values might indicate a corresponding increase in the number of patients showing false positive BT responses, while at the higher cut-off SI values, it might be anticipated that a correspondingly increased number of patients would show false negative BT responses. In the subsequent analysis, therefore, SI $\geqslant 1 \cdot 6$ was scored as a positive BT response.

The *in vitro* CMI responses to PPD and the extracts, by the NPC and the OC patients are compared (Fig. 3). The BT responses to PPD and the NPC biopsy extract, 2PE, were not significantly different between the 2 groups of patients, but a significantly greater number of NPC patients displayed positive responses to TS, ES and 12PE. The 2 groups of patients were not significantly different in their MIF responses to PPD but significantly more NPC than OC patients showed positive MIF response to TS, ES, 2PE and 12PE. Among the concordant responders, the 2 groups of patients differed significantly in their responses to all 4 extracts but not to PPD. The discordant responses were probably due either to a weak CMI of the patients tested or to inherent artifacts of the assays. In the first instance, it might be anticipated that the results obtained by both assays were random, depending on the relative sensitivity of the assays. In the latter instance, as both assays differ in nature, assay artifacts may be expected to become apparent under different sets of experimental conditions and thereby giving rise to discordant responses. However, these possibilities are not readily distinguishable from one another, and patients who showed discordant responses were excluded from consideration. The number of NPC and OC patients thus excluded from the top section of Fig. 3, were 10/44 tested for their responses against PPD, 9/39 for TS, 15/38 for ES, 9/30 for 2PE and 7/30 for 12PE. (These can

TABLE III.—*Subjects Tested on BT Assay Only*

| Subjects | Number Male | Female | Total | Mean age ± s.d. (yrs) |
|---|---|---|---|---|
| NPC patients (Stages I and II) | 17 | 3 | 20 | 45·2±9·3 |
| NPC patients (Stages III and IV) | 44 | 16 | 60 | 48·1±10·1 |
| *Other cancer patients (OC) | 42 | 15 | 57 | 57·0±10·6 |
| Healthy subjects (HS) | 15 | 6 | 21 | 38·8±11·2 |

* The OC patients were made up of 15 with Ca bronchus, 11 Ca larynx, 4 Ca tongue, 2 each with Ca cervix, oesophagus and basal cells, 2 with malignant thymoma, 3 with lymphoma, 1 each with Ca of hard palate, soft palate, hypopharynx, maxillary antrum, parotid gland, epiglottis, maxillary sinus, testis, urinary bladder, supraglottis, vocal cord, bone axilla and 1 patient each with Hodgkin's disease, metastatic carcinoma and fibrosarcoma.

TABLE IV.—*Comparison of the Blastogenic Responses shown by Leucocytes from Other Cancer Patients (OC) and Healthy Subjects (HS) Cultured in the Presence of PPD, the Raji-cell Extracts, TS and ES, or the NPC-biopsies Extracts, 2PE and 12PE*

| | OC | | HS | | Comparison (*P*) | |
|---|---|---|---|---|---|---|
| Antigens | No. | Median SI | No. | Median SI | Wilcoxon | Chi-square |
| PPD | 57 | 1·5 | 13 | 2·0 | ns | ns |
| TS | 51 | 0·9 | 21 | 1·0 | ns | ns |
| ES | 51 | 0·8 | 21 | 0·8 | ns | ns |
| 2PE | 57 | 1·4 | 20 | 1·1 | ns | ns |
| 12PE | 56 | 1·2 | 20 | 0·9 | ns | ns |

be seen as categories in C and D in Fig. 1.)

In a more extensive study, involving a large number of NPC and OC patients and HS, Table III, only the BT assay was used. The results thus obtained were analysed by the Wilcoxon's two-sample rank-sum test as well as by the Chi-square-test using SI = 1·6 as the cut-off value. Neither method of comparison revealed a significant difference between OC and HS in the distribution of SI with PPD or the extracts. These 2 groups were, therefore, combined as the control groups (Table IV). NPC patients as a whole, or those with regional tumours, differed significantly from the controls in their BT responses to TS, ES, 2PE and 12PE, but not to PPD, as indicated by both methods of comparison (Table V(*a*) and (*b*)). NPC patients with local tumours, however, showed similar responses to PPD, 2PE and 12PE, but the responses to TS and ES differed significantly between these 2 groups (Table V(*c*)). These results suggest therefore that the BT responses, at least to the biopsy extracts, may be more strongly associated with the later stages of NPC.

### DISCUSSION

The blast transformation (BT) assay, particularly when adapted for micro-cultures, provides a convenient means of testing for *in vitro* CMI, but the evaluation of data so obtained presents certain difficulties. One concerns the different levels of spontaneous activity of the control unstimulated cultures, which make it necessary, as in the present instance, to measure BT responses in terms of SI rather than the absolute amount of radioactivity incorporated. The choice of a cut-off value to score BT responses presents yet another difficulty. To apply this assay to the evaluation of the CMI of an individual against a known antigen, a large-scale population study should first be carried out. From the results thus obtained, density functions of SI's from a sensitized and non-sensitized populations may be generated. An appropriate cut-off value of SI may

TABLE V.—*Comparison of the Blastogenic Responses shown by Leucocytes from NPC Patients and Control Subjects (C) which Included Patients with Other Cancers (OC) and Healthy Subjects (HS) Cultured in the Presence of PPD, the Raji-cell Extracts, TS and ES, or the NPC-biopsies Extracts, 2PE and 12PE*

(*a*) NPC *vs* C

| | NPC | | C | | Comparison (*P*) | |
|---|---|---|---|---|---|---|
| Antigens | No. | Median SI | No. | Median SI | Wilcoxon | Chi-square |
| PPD | 80 | 2·0 | 70 | 1·7 | ns | ns |
| TS | 69 | 1·4 | 72 | 0·9 | 0·0002 | 0·01 |
| ES | 68 | 1·3 | 72 | 0·8 | 0·0001 | 0·01 |
| 2PE | 56 | 2·2 | 77 | 1·3 | 0·0005 | 0·01 |
| 12PE | 56 | 1·7 | 76 | 1·2 | 0·0014 | 0·05 |

(*b*) NPC stages III and IV *vs* C

| | NPC III and IV | | C | | Comparison (*P*) | |
|---|---|---|---|---|---|---|
| Antigens | No. | Median SI | No. | Median SI | Wilcoxon | Chi-square |
| PPD | 60 | 1·9 | 70 | 1·7 | ns | ns |
| TS | 53 | 1·4 | 72 | 0·9 | 0·0005 | 0·01 |
| ES | 53 | 1·3 | 72 | 0·8 | 0·0001 | 0·01 |
| 2PE | 43 | 2·4 | 77 | 1·3 | 0·0001 | 0·01 |
| 12PE | 43 | 1·8 | 76 | 1·2 | 0·0002 | 0·05 |

(*c*) NPC stages I and II *vs* C

| | NPC I and II | | C | | Comparison (*P*) | |
|---|---|---|---|---|---|---|
| Antigens | No. | Median SI | No. | Median SI | Wilcoxon | Chi-square |
| PPD | 20 | 2·0 | 70 | 1·7 | ns | ns |
| TS | 16 | 1·4 | 72 | 0·9 | 0·0088 | ns |
| ES | 15 | 1·2 | 72 | 0·8 | 0·0001 | 0·01 |
| 2PE | 13 | 1·6 | 77 | 1·3 | ns | ns |
| 12PE | 13 | 1·4 | 76 | 1·2 | ns | ns |

then be chosen on the basis of such distribution patterns, but the diagnostic value of such tests will depend on the separation between the density functions. In the case of a heterogeneous mixture of unknown antigenicity, such as the extracts used for the present study, it may not be possible to decide on an objective cut-off value which delineates the sensitized and non-sensitized subjects. As our present interest lies, however, in differences between the NPC patients and the control group, it is not essential that such a cut-off value be established. Instead, the distribution of SI values with a given antigen preparation in 2 groups of subjects may be directly compared by the Wilcoxon's two-sample rank-sum test. Such differences may also be reflected in the different frequencies of individuals from the 2 groups having an SI exceeding or equal to a certain arbitrarily chosen value. Such an arbitrary cut-off value may be chosen empirically, because it gives the best discrimination between the 2 groups of subjects tested. In the present instance, it was chosen as a value which gave an optimal concordance between the results of the BT and MIF assays. If the discordance is due to assay artifacts, as discussed earlier, this value would approximate the true cut-off value. Valdimarsson *et al.* (1972) reported that lymphocytes from a severe chronic case of mucocutaneous candidiasis of the granulomatous variety, responded to *in vitro* challenge with soluble candida antigens by elaboration of MIF but not by increased DNA synthesis. Eife *et al.* (1974)

also found discordant BT responses and lymphotoxin production in neonatal lymphocytes. It seems possible, therefore, that the present discordance may reflect an inherent characteristic in the immunity of some of the subjects. Other possibilities giving rise to assay discordance may include toxicity of the antigen preparations, as suggested by the low median SI values observed with TS and ES. In the latter instance, it is necessary to assume that antigen toxicity affects the two assays differently.

It must be emphasized that the cut-off value as used herein is arbitrarily chosen to allow group comparison, and it is not intended for testing *in vitro* CMI of individual subjects. To facilitate the differentiation of individual NPC patients and control subjects, Professor John Aitchinson has carried out a parametric analysis of the present data, in which density function of SI values obtained with all 5 antigen preparations were generated simultaneously for the NPC and the non-NPC groups. BT responses to the 5 antigen preparations observed in individual subjects were then compared to these 2 density functions. It was found that about 80% of the control subjects show response profiles which fit in with the density functions for the non-NPC group, whilst about 50% of the NPC patients have an "NPC-like" pattern of responses.

The comparisons of the *in vitro* CMI responses of NPC patients and control subjects to PPD and the extracts of Raji cells and NPC biopsies are summarized in Table VI. It is apparent that the NPC patients and the control subjects do not differ in their responses to PPD. By contrast, the responses elicited by the Raji-cells and the NPC-biopsy extracts appear to be associated with NPC. Thus, a significantly greater number of NPC than OC patients showed positive MIF responses to all 4 extracts, and positive BT responses to only the Raji-cell extracts. Among the concordant responders, a preponderance of the NPC patients showed positive MIF and BT responses to all 4 extracts, while the OC patients almost uniformly showed a negative response. In the larger series using the BT assay alone, the distribution patterns of SI values observed with the NPC patients and the control subjects using these extracts were found to be significantly different by Wilcoxon's two-sample rank-sum test. There was also a significantly greater number of NPC patients than control subjects showing positive BT responses (SI $\geqslant$ 1·6) to each of the 4 extracts. A positive response to the biopsy extracts (2PE and 12PE) appeared

TABLE VI.—*Comparison of* In vitro *CMI Responses between NPC Patients and Control Groups, Patients with Other Cancer (OC) or OC + HS (C)*

| *In vitro* CMI assay | Groups compared | Method of comparison | Antigens (*P*) PPD | TS | ES | 2PE | 12PE |
|---|---|---|---|---|---|---|---|
| BT | (1) NPC *vs* OC | *SI $\geqslant$ 1·6 | †ns | <0·01 | <0·01 | ns | <0·02 |
| | (2) NPC *vs* C | SI $\geqslant$ 1·6 | ns | <0·01 | <0·01 | <0·01 | <0·05 |
| | NPC *vs* C | Wilcoxon | ns | 0·0002 | 0·0001 | 0·0005 | 0·0014 |
| | (3) NPC (I and II) *vs* C | SI $\geqslant$ 1·6 | ns | ns | <0·01 | ns | ns |
| | NPC (I and II) *vs* C | Wilcoxon | ns | 0·0088 | 0·0001 | ns | ns |
| | (4) NPC (III and IV) *vs* C | SI $\geqslant$ 1·6 | ns | <0·01 | <0·01 | <0·01 | <0·05 |
| | NPC (III and IV) *vs* C | Wilcoxon | ns | 0·0005 | 0·0001 | 0·0001 | 0·0002 |
| MIF | (5) NPC *vs* OC | *% migration $\leqslant$ 75 | ns | <0·01 | <0·01 | <0·01 | <0·01 |
| MIF/BT | (6) NPC *vs* OC | *% migration $\leqslant$ 75 and SI $\geqslant$ 1·6 | ns | <0·01 | <0·01 | <0·01 | <0·01 |

* Chi square analysis using SI $\geqslant$ 1·6 or average percent migration $\leqslant$ 75 as the appropriate cut-off values.
† Not significant.

more frequently at the later stage of the disease.

The present observations with the Raji-cell extracts are consistent with the findings of Levine *et al.* (1976) and Ho *et al.* (1977*b*). Both groups of authors observed that there was a significantly greater number of NPC patients than control subjects who showed positive skin reactivity to the membrane extract of the EBV-carrying lymphoblastoid cell line, HKLy28. In a longitudinal study of NPC patients following radiation therapy (RT), Ho *et al.* (1977*b*) observed 2 predominant patterns of changes in the skin reactivity to the HKLy28 extracts. A positive initial skin reaction to the HKLy28 extract, becoming negative after RT, was frequently associated with a good prognosis, while a preponderance of NPC patients with a bad prognosis after RT showed persistence of a positive skin reaction throughout the period of observation. Our observations with the biopsy extracts were also in harmony with the results of Chu *et al.* (1967) which suggested the existence of NPC-related CMI. The antigenic specificity of the NPC associated CMI observed with both types of extract is of obvious interest. However, as the extracts thus far used to demonstrate such CMI status were heterogeneous mixtures, the question of antigenic specificity must therefore await studies (now in progress) using purified fractions of these extracts. We are also testing similar extracts of the EBV-negative lymphoblastoid BJAB cells, and of biopsy specimens obtained from the primary tumour sites of patients with other cancers, in order to further characterize the NPC-associated CMI observed with our present extracts and to see whether it is NPC- or EBV-related or both.

The present observations with PPD accord with those of Ho *et al.* (1977) who used extracts of trichophyton and monilia as the recall antigens. In both instances, no apparent differences were observed between the CMI responses of NPC and control subjects to these recall antigens. Chan *et al.* (1976) however, observed that the responses of peripheral leucocytes from NPC patients to PHA stimulation and their skin reactions to PPD were less than those from control subjects. The reason for such apparent discrepancy remains to be resolved.

This work was supported by a research contract from the International Agency for Research on Cancer and by grants from the Hong Kong Anti-cancer Society and the Hong Kong University Committee for Higher Education and Research. We are most grateful to Professor J. Aitchinson for his interest and advice on statistical analysis of data.

### REFERENCES

CHAN, S. H., CHEW, T. S., GOH, E. H., SIMONS, M. J. & SHANMUGARATNAM, K. (1976) Impaired General Cell-mediated Immune Function *In vivo* and *In vitro* in Patients with Nasopharyngeal Carcinoma. *Int. J. Cancer*, **18**, 139.

CHU, E. H. Y., STJERNSWARD, J., CLIFFORD, P. & KLEIN, G. (1967) Reactivity of Human Lymphocytes Against Autochthonous and Allogeneic Normal and Tumor Cells *In vitro*. *J. natn. Cancer Inst.*, **39**, 595.

DE SCHRYVER, A., KLEIN, G., HENLE, W. & HENLE, G. (1974) EB Virus-associated Antibodies in Caucasian Patients with Carcinoma of the Nasopharynx and in Long-term Survivors after Treatments. *Int. J. Cancer*, **13**, 319.

DESGRANGES, C., WOLF, H., DE-THÉ, G., SHANMUGARATNAM, K., ELLOUZ, R., CAMMOUN, N., KLEIN, G. & ZUR HAUSEN, H. (1975) Nasopharyngeal Carcinoma. X. Presence of Epstein–Barr Genomes in Epithelial Cells of Tumours from High and Medium Risk Areas. *Int. J. Cancer*, **16**, 7.

DE-THÉ, G., HO, J. H. C., ABLASHI, D. V., DAY, N. E., MACARIO, A. L., MARTIN-BERTHELON, M. C., PEARSON, G. & SOHIER, R. (1975) Nasopharyngeal Carcinoma. IX. Antibodies to EBNA and Correlation with Response to other EBV Antigens in Chinese Patients. *Int. J. Cancer*, **16**, 713.

EIFE, R. D., EIFE, G., AUGUST, C. S., KUHRE, W. L. & STAEHR-JOHANSEN, K. (1974) Lymphotoxin Production and Blast Cell Transformation by Cord Blood Leukocytes, Dissociated Lymphocyte Function in New Born Infants. *Cell. Immunol.*, **14**, 435.

GERBER, P. (1972) Activation of Epstein–Barr Virus by 5-Bromodeoxyuridine in Virus-free Human Cells. *Proc. natn. Acad. Sci., U.S.A.*, **69**, 83.

GLASER, R., DE-THÉ, G., LENOIR, G. & HO, J. H. C. (1976) Superinfection of Epithelial Nasopharyngeal Carcinoma Cells with Epstein–Barr Virus. *Proc. natn. Acad. Sci., U.S.A.*, **73**, 960.

HABEL, K. (1969) Antigens of Virus Induced Tumors. *Adv. Immunol.*, **10**, 229.

HAMPAR, B., DERGE, J. G., MARTOS, L. M., TAGAMETS, M. A., CHANG, S. Y. & CHAKRABARTY, M. (1973) Identification of a Critical Period during the S-Phase for Activation of the Epstein–Barr Virus by 5-Iododeoxyuridine. *Nature, New Biol.*, **244**, 214.

HENLE, G., HENLE, W. & KLEIN, G. (1971) Demonstration of Two Distinct Components in the Early Antigen Complex of EBV Infected Cells. *Int. J. Cancer*, **8**, 272.

HENLE, W., HENLE, G., BURTIN, P., CACHIN, Y., CLIFFORD, P., DE SCHRYVER, A., DE-THÉ, G., DIEHL, V., HO, H. C. & KLEIN, G. (1970) Antibodies to Epstein–Barr Virus in Nasaopharyngeal Carcinoma, Other Head and Neck Neoplasms and Control Groups. *J. natn. Cancer Inst.*, **44**, 225.

HENLE, W., HO, H. C., HENLE, G. & KWAN, H. C. (1973) Antibodies to Epstein–Barr Virus Related Antigens in Nasopharyngeal Carcinoma. Comparison of Active Cases and Long-term Survivors. *J. natn. Cancer Inst.*, **51**, 361.

HO, H. C., NG, M. H., KWAN, H. C., CHAN, J. C. W. (1976) Epstein–Barr Virus-specific IgA and IgG Serum Antibodies in Nasopharyngeal Carcinoma Patients and Controls. *Br. J. Cancer*, **34**, 655.

HO, H. C., CHAN, J. W. C., NG, M. H. & LEVINE, P. H. (1977*a*) *In vivo* CMI Studies in Nasopharyngeal Carcinoma to EBV Related and Unrelated Antigens. In *International Symposium on Etiology and Control of Nasopharyngeal Carcinoma*. Kyoto, Japan, (in press).

HO, H. C., NG, M. H., KWAN, H. C. (1977*b*) IgA Antibodies to Epstein–Barr Viral Capsid Antigens in Saliva of Nasopharyngeal Carcinoma Patients. *Br. J. Cancer*, **35**, 888.

HUANG, D., HO, J. H. C., HENLE, W. & HENLE, G. (1974) Demonstration of Epstein–Barr Virus-associated Nuclear Antigen in Nasopharyngeal Carcinoma Cells from Fresh Biopsies. *Int. J. Cancer*, **14**, 580.

HUANG, D. P., HO, H. C., NG, M. H., LUI, M. (1977) Possible Transformation of Nasopharyngeal Epithelial Cells in Culture with Epstein–Barr Virus Derived from B95–8 Cells. *Br. J. Cancer*, **35**, 360.

KLEIN, G., GIOVANELLA, B., LINDAHL, T., FIALKOW, P. J., SINGH, S. & STEHLIN, J. (1974) Direct Evidence for the Presence of Epstein–Barr Virus DNA and Nuclear Antigen in Malignant Epithelial Cells from Patients with Anaplastic Carcinoma of the Nasopharynx. *Proc. natn Acad. Sci., U.S.A.*, **71**, 4737.

LAI, P. K., ALPERS, M. P., MACKAY-SCOLLAY, E. M. (1975) *In vitro* Evaluation of Cell Mediated Immunity to Epstein–Barr Herpesvirus by Cell Migration Inhibition Tests. *J. natn. Cancer Inst.*, **55**, 1319.

LEVINE, P. H., DE-THÉ, G. B., BRUGERE, J., SCHWAAB, G., MOURALI, N., HERBERMAN, R. B., AMBROSIONI, J. C. & REVOL, P. (1976) Immunity to Antigens Associated with Cell Line Derived from Nasopharyngeal Cancer (NPC) in Non-Chinese NPC Patients. *Int. J. Cancer*, **17**, 155.

LO, E. H. M., NG, M. H., NG, W. S. & HO, H. C. (1976) Studies on the Polypeptides Solubilized by Ionic Shock Treatments of Raji Cells by Acrylamide Gel Electrophoresis and Lactoperoxidase Radioiodination. *Biochim. biophys. Acta*, **444**, 863.

NKRUMAH, F., HENLE, W., HENLE, G., HERBERMAN, R., PERKINS, V. & DEPUR, R. (1976) Burkitt's Lymphoma: its Clinical Course In Relation to Immunologic Reactivities to Epstein–Barr Virus and Tumor-related Antigens. *J. natn. Cancer Inst.*, **57**, 1051.

NG, M. H., HO, H. C. & KWAN, H. C. (1977) Genetic and Antigenic Basis for the IgA Antibody Response to VCA. In *International Symposium on Etiology and Control of Nasopharyngeal Carcinoma*. Kyoto, Japan, (in press).

REEDMAN, B. M. & KLEIN, G. (1973) Cellular Localization of an Epstein–Barr Virus (EBV) Associated Complement-fixing Antigen in Producer and Non-producer Lymphoblastoid Cell Lines. *Int. J. Cancer*, **11**, 499.

ROCKLIN, P. E., MEYERS, O. L. & DAVID, J. R. (1970) An *In vitro* Assay for Cellular Hypersensitivity in Man. *J. Immunol.*, **104**, 95.

SVEDMYR, E. & JONDAL, M. (1975) Cytotoxic Effector Cells Specific for B Cell Lines Transformed by Epstein–Barr Virus Are Present in Patient with Infectious Mononucleosis. *Proc. natn. Acad. Sci., U.S.A.*, **72**, 1622.

TRUMPER, P. A., EPSTEIN, M. A. & GIOVANELLA, B. C. (1976) Activation *In vitro* by BUdR of a Productive EB Virus Infection in the Epithelial Cells of Nasopharyngeal Carcinoma. *Int. J. Cancer*, **17**, 578.

VALDIMARSSON, H., WOOD, C. B. S., HOBBS, J. R. & HOLT, P. J. L. (1972) Immunological Features in a Case of Chronic Granulomatous Candidiasis and its Treatment with Transfer Factor. *Clin. exp. Immunol.*, **11**, 151.

WOLF, H., ZUR HAUSEN, H. & BECKER, V. (1973) EB Viral Genomes in Epithelial Nasopharyngeal Carcinoma Cells. *Nature, New Biol.*, **244**, 245.